# Estimation of the thermocapillary force and its applications to precise droplet control on a microfluidic chip

**DOI:** 10.1038/s41598-017-03028-w

**Published:** 2017-06-08

**Authors:** By June Won, Wooyoung Lee, Simon Song

**Affiliations:** 1 Dept. of Mechanical Engineering, Hanyang University, Seoul, 04763 Korea; 2 Insitute of Nano Science and Technology, Hanyang University, Seoul, 04763 Korea

## Abstract

Droplet control through the use of light-induced thermocapillary effects has recently garnered attention due to its non-intrusive and multifunctional nature. An important issue in droplet control is the estimation of the thermocapillary force. The purpose of the present study is to estimate the thermocapillary force and propose empirical equations between the force and simply measurable key parameters such as droplet diameter and power of heat source. In addition, we aim to shift the droplet trajectory and develop an on-demand droplet routing system based on the estimation of the thermocapillary force. We illuminated a continuous phase with a 532 nm laser beam to minimize possible damage or property changes to target molecules contained within droplets. A mixture of light-absorbing material and oleic acid was used for the continuous phase fluid, while deionized water (DI water) was used for the dispersed phase fluid. We proposed empirical equations to estimate the thermocapillary force, which was then applied to precise droplet shifting and routing. We found that the shifting distance was linearly proportional to the thermocapillary force, and that an on-demand droplet routing system resulted in a success rate greater than 95%.

## Introduction

Droplet control is one of the most important techniques in the field of microfluidics. A bio-sample contained within a droplet can be conveyed from one spot to another without contamination or can be rapidly mixed with reactants to perform a biochemical assay. Additionally, droplets containing various samples can be sorted or split via droplet control techniques. Due to these merits, many studies have been performed with regard to droplet control^1^. For instance, passive methods utilizing channel geometry^2–4^, magnetic forces^5–7^, electrowetting-on-dielectrics (EWOD)^8–10^, optical tweezers^11–13^, and Marangoni effects^14–16^ have been presented as means of droplet control.

Optical droplet control methods have garnered attention due to their nonintrusive nature. Furthermore, a multifunctional droplet control system can be readily developed because only the light patterns need to be changed to vary the function^17–19^. Optical droplet control methods are categorized as either photon momentum methods or interfacial gradient methods^20^. The former includes the use of radiation pressures^21, 22^ and optical tweezers^11–13^, while the latter utilizes the generation of wettability gradients^23, 24^ or interfacial tension gradients, i.e., light-driven Marangoni effects^14, 15, 25^.

Marangoni effects, which were first explained by Thomson^26^, occur when the interfacial tension along the interface between two media is different. As a result, a secondary flow is generated from a lower-interfacial tension area to a higher-interfacial tension area. Depending on the cause of the interfacial tension difference, these effects are categorized into thermocapillary effects utilizing a temperature gradient, solutalcapillary effects utilizing a surfactant concentration difference, and chromocapillary effects utilizing photosensitive surfactants and UV light^20, 27^. Among these, thermocapillary effects can be subdivided into two types based on the sign of the interfacial tension (γ) change as a function of temperature (T): attractive thermocapillary effects (i.e., the droplet moves toward a higher- temperature region) when the interfacial tension decreases with increasing temperature (dγ/dT < 0) and repulsive thermocapillary effects (i.e., the droplet moving toward a lower- temperature region) in the opposite case (dγ/dT > 0). In general, for most fluids, attractive thermocapillary effects occur when the molecular cohesion forces decrease with an increase in temperature. However, the interfacial tension of a mixture solution may increase with an increase in temperature due to the presence of neutral surfactants. This results in an increase in the molecular cohesion due to chemical characteristics^28^; consequently, repulsive thermocapillary effects are observed.

In 1959, Young *et al*. experimentally demonstrated thermocapillary effects for the first time^29^. The position of a bubble was fixed due to the force equilibrium between buoyancy and the thermocapillary force. In 2003, Garnier *et al*. demonstrated light-induced thermocapillary effects in an open channel^30^, in which a light-absorbing substrate was placed on the bottom. When light illuminated the free surfaces in the channel, the liquid was attracted toward the light. In 2004, Rybalko *et al*. successfully controlled the position of an oil droplet floating on water by using attractive thermocapillary effects^31^. In 2016, Mutto *et al*. investigated the effects of oil droplet size and temperature difference around droplet on the deflection of droplet, and they also estimated the thermocapillary force as a function of the distance to the laser spot^32^. In contrast, Faris *et al*. found that a water droplet could be pushed away from a laser beam in an open channel^33^. By illuminating droplets with a laser beam, Baroud *et al*. applied repulsive thermocapillary effects toward various droplet control functions such as microfluidic valves, droplet fusion, division, and sorting^34^.

Most of these studies generated thermocapillary effects by directly heating the droplet^25, 27, 31, 33–35^. As a result, bio-samples within a droplet would be likely to be damaged due to direct heating. A method to minimize this damage is to heat the continuous phase instead of the droplets and push a droplet away from a light source via repulsive thermocapillary effects. Some studies have adapted this method; however, these have been limited to open channels^14^ or oil droplets^32^ instead of water droplets. Furthermore, there are few studies that have estimated the thermocapillary forces acting on a droplet^32, 36–38^. According to these studies, temperature distribution around a droplet is required for the force estimation. However, it is hard to measure the distribution especially for the case of using an opaque fluid in a closed channel. Thus, a relation between the force and simply measureable parameters such as droplet diameter, laser power, etc. needs to be derived.

In this study, we investigate the relationships between thermocapillary force and key parameters such as power of the heat source and droplet diameter. The magnitude of thermocapillary force is estimated by assuming the formation of a quasi-equilibrium state during droplet control. Parameter studies are performed to examine the effects of power of the heat source and droplet diameter on thermocapillary force. Specifically, we propose an empirical equation for the thermocapillary force, power of the heat source, and droplet size. We also examine the relation between droplet shifting distance and thermocapillary force. Finally, the performance of the on-demand droplet routing system is examined.

## Results and Discussion

### Estimation of the thermocapillary force

**Figure 1:**
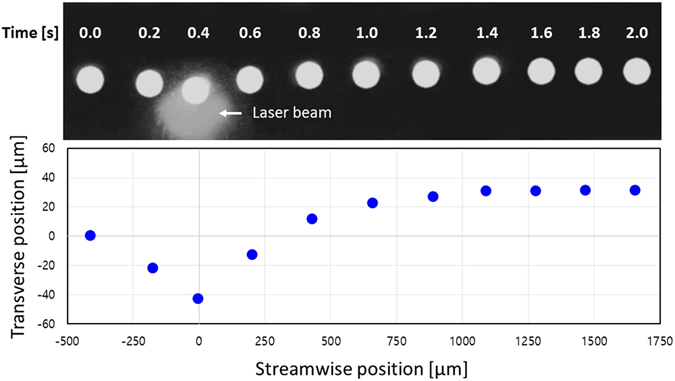
Trajectory shifting due to repulsive thermocapillary effects. The upper image was overlapped with 11 successive images captured at 0.2 s intervals. The lower graph shows the corresponding droplet trajectories. The streamwise position was set to zero at the laser beam center, and the transverse position was set to zero at the centerline of the channel. The shifting distance was defined as the difference in transverse positioning of the droplet between downstream and upstream. In this graph, the shifting distance was about 31.3 μm. The streamwise velocity of the droplet was 1.16 mm/s, and the irradiated laser power was 70 mW.

We identified repulsive thermocapillary effects with a water droplet in an ink mixture prior to estimating the thermocapillary force. First, we found that the viscosity exponentially decreased with increasing temperature (Figure S1a)^39^. Next, the interfacial tension (*γ*) between DI water and the ink mixture increased with an increase in temperature (*T*), as shown in Figure S1b, where *dγ*/*dT* was positive. The value of *dγ*/*dT* was 0.0328 mN∙m^−1^°C^−1^ for the mixture. This means that a repulsive thermocapillary flow occurs if there is local heating. As shown in Fig. 1, the trajectory of the droplet (95 μm in diameter) shifted away from the center of the laser beam. Therefore, it is obvious that repulsive thermocapillary effects occurred due to the temperature gradient generated by the laser beam. Interestingly, the droplet moved slightly toward the laser beam and was then pushed away downstream. This was because the continuous phase fluid was heated locally, causing its viscosity to decrease, which led to a streamline curvature toward the local temperature maxima (i.e., the laser beam center). It is well known that fluid velocity is inversely proportional to viscosity. Thus, a local viscosity decrease results in a local increase in fluid velocity, a subsequent pressure drop, and streamline curvature (Figure S2).

According to the Marangoni number, defined as the ratio of surface tension to viscous force^40^,

1 $$Ma=-\frac{d\gamma }{dT}\frac{D\,{\rm{\Delta }}T}{{\mu }_{o}\alpha }$$

the key parameters affecting the thermocapillary force are droplet diameter (D), temperature difference (ΔT) across the droplet, and viscosity (*μ*_*o*_) of the continuous phase fluid. The dγ/dT term was considered to be a constant (Figure S1b). The thermal diffusivity (α) can also be considered as a constant because the measured value hardly changed, regardless of temperature variation. For example, the thermal diffusivity values were 0.098, 0.095, and 0.096 mm^2^/s at 25, 50, and 75 °C, respectively. Therefore, only three variables (*D*, ΔT, and *μ*_*o*_) affect the thermocapillary force. Thus, we selected droplet diameter and power of the heat source as key parameters because both ΔT and *μ*_*o*_ strongly depend on the power of the heat source, which is provided by the laser beam.

**Figure 2:**
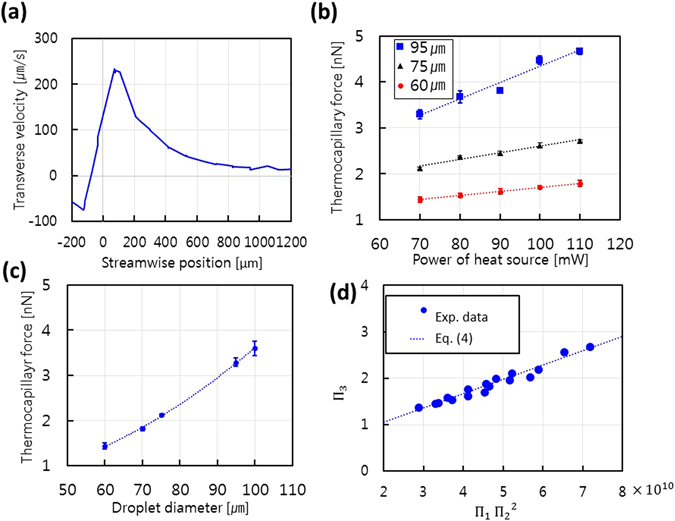
The thermocapillary force estimation and its empirical relations. (**a**) The transverse velocity of a droplet with a diameter of 60 μm and a streamwise velocity of 1.16 mm/s. The velocity was estimated via images successively captured at 30 fps. The transverse velocity reached its maximum at a point about 100 μm downstream from the laser beam center. The effects of (**b**) power of the heat source (linear fitting, R^2^ = 0.9606) and (**c**) droplet diameter (square fitting, R^2^ = 0.9993) on thermocapillary force. Each point was obtained by repeating the experiment 10 times. (**d**) The non-dimensional relationships of thermocapillary force (Π_3_) to power of the heat source (Π_1_) and droplet diameter (Π_2_). The dotted lines represent Equation (4) with coefficients A_1_ and A_2_ equal to 3.088 × 10^−11^ and 0.432, respectively. The R^2^ value is 0.957.

To estimate the thermocapillary forces exerted on the droplets, we assumed that there was a moment where a quasi-equilibrium state existed between the drag force and the thermocapillary force in the microchannel. Figure 2a displays the transverse velocity variation of droplets caused by the repulsive thermocapillary forces for a laser power of 70 mW. The velocity rapidly increased when passing by the center of the laser beam and gradually decreased afterward. Therefore, the quasi-equilibrium assumption was valid when the velocity reached its maximum. On the other hand, the drag force on a spherical droplet within a rectangular channel was calculated with Equation 2 ^41^ because the droplet sizes (95 μm) in this experiment were smaller than the channel height (100 μm):

2 $${F}_{drag}=3\pi D{\mu }_{o}U\frac{1+2{\mu }_{o}/3{\mu }_{i}}{1+{\mu }_{o}/{\mu }_{i}}$$

where D is droplet diameter, *μ*_*i*_ is the viscosity of the dispersed phase fluid, and *U* is the transverse velocity of the droplet. We found that the term (1 + 2*μ*_*o*_/3*μ*_*i*_)/(1 + *μ*_*o*_/*μ*_*i*_) was almost independent of temperature for the given conditions (see Supplementary Information). Thus, measuring the maximum transverse velocity led to an estimation of the repulsive thermocapillary force (transverse component) according to the quasi-equilibrium assumption.

Figure 2b shows the effects of laser power on thermocapillary force. The laser power increased from 70 to 110 mW, and the droplet diameter was varied from 60 to 95 μm, maintaining a droplet velocity of 1.13 ± 0.03 mm/s. The thermocapillary force increased linearly with an increase in laser power for the given droplet sizes; this is in good agreement with previous studies^42^. These results can be explained by the relationship between *Ma* and Δ*T* in Equation (1). The laser power absorbed into the thermal energy of the continuous phase contributed to an increase in Δ*T*. Also, the viscosity decreased with an increase in temperature (Figure S1a). Therefore, the thermocapillary effects were enhanced as the laser power increased.

Figure 2c shows the effect of droplet diameter on thermocapillary force. The droplet size was varied from 60 to 100 μm, while the droplet velocity and laser power were fixed at 1.13 mm/s and 70 mW, respectively. Unlike the laser power, the force non-linearly increased as the droplet diameter increased. The curve was fit better with a square (R^2^ = 0.9993) curve than a linear line (R^2^ = 0.9942). The slopes of data in Fig. 2b also supported this argument. These values were 0.0088, 0.0144, and 0.0354 nN/mW for droplet sizes of 60, 75, and 95 μm, respectively. If the effects of droplet size on the force were linear, the slope should be constant regardless of droplet size. Furthermore, the Marangoni number also implies that the effects are non-linear. Under the same conditions (excepting the droplet size), a large droplet (*a*) is subject to a higher temperature difference (ΔT) across the droplet. Thus, the Marangoni effect is simultaneously affected by both droplet size and temperature difference, even when only the droplet size is varied. This implies that a temperature gradient is constant around a droplet in a quasi-equilibrium state where it is located near the edge of laser beam as shown in Fig. 1. Note that Maria *et al*. also showed a dramatic but linear temperature decrease near the laser beam edge^43^.

The estimated thermocapillary forces are between 1 nN and 5 nN for a droplet that is 60 to 100 μm in diameter; these values are similar to those found in the literature. For example, Muto *et al*. showed that their maximum force was about 7.2 nN for a spherical droplet with a diameter of 77 μm^32^. Verneuil *et al*. reported that the force acting on a slug droplet, with a major radius of about 250 μm, was 100 nN^27^. Thus, we believe that using the quasi-equilibrium assumption provides reasonable results when estimating thermocapillary forces.

### Empirical formulation for thermocapillary force

Based on our detailed parameter studies investigating thermocapillary force, we propose empirical relationships between thermocapillary force and some key parameters. These have been non-dimensionalized by applying Buckingham’s Pi theorem^41^, as follows:

3 $${{\Pi }}_{1}=\frac{\,P}{{\mu }_{o}\,D\,{V}^{2}},{{\Pi }}_{2}=\frac{D\,V}{\alpha },{{\Pi }}_{3}=\frac{F}{D\,V\,{\mu }_{o}}$$

*∏*_1_, *∏*_2_, and *∏*_3_ represent the dimensionless power of the heat source, droplet diameter, and thermocapillary force, respectively. The variables D, P, F, and V indicate droplet diameter, power of the heat source (laser beam power), thermocapillary force, and droplet velocity, respectively. We included velocity in the non-dimensionalization because it affects the temperature distribution via convective heat transfer. Interestingly, *∏*_2_ has the form of a Peclet number. We already showed that the thermocapillary force is linearly proportional to power of the heat source and proportional to the square of droplet diameter. Therefore, their relationships can be represented with dimensionless variables, as follows:

4 $${{\Pi }}_{3}={{\rm{A}}}_{1}\cdot {{\Pi }}_{1}\cdot {{{\Pi }}_{2}}^{2}+{A}_{2}$$

The coefficient A_1_ and A_2_ can be determined from the experimental results. Figure 2d shows that the empirical relations for thermocapillary force are in good agreement with the experimental results. This implies that our proposed relationship between thermocapillary force and droplet size and power of the heat source is reasonable.

The proposed empirical relation is compatible with the thermocapillary model suggested by Gallarie *et al*.^42^ although heir model was developed for a pancake–shaped droplet in a Hele-Shaw cell. They proposed that the magnitude of the thermocapillary force can be expressed as follows:

5a $${\rm{F}}={\rm{\pi }}\,{\rm{a}}\,{\rm{D}}\,{\rm{\Delta }}T\,\frac{{\mu }_{o}}{{\mu }_{o}+{\mu }_{i}}\,\frac{d\,\gamma }{d\,T}$$

Here, “a” represents the aspect ratio between channel height and droplet diameter, which is a constant for a given droplet size. Also, the term $\frac{{\mu }_{o}}{{\mu }_{o}+{\mu }_{i}}\,\frac{d\,\gamma }{d\,T}$ is a constant, enabling Equation (5a) to be rewritten as

5b $${\rm{F}}={{\rm{A}}}_{3}\,{\rm{D}}\,{\rm{\Delta }}T$$

Note that we argued that the temperature difference Δ*T* depends on droplet diameter and power of the heat source in the discussion of Fig. 2. Thus, Equation (5b) can be expressed as

5c $${\rm{F}}={{\rm{A}}}_{3}\,{\rm{D}}\,f(D,P)$$

where Δ*T* was replaced by a function of droplet diameter (D) and power of the heat source (P). Comparing Equation (5c) with a dimensional form of Equation (4), which is given as

6 $${\rm{F}}={{\rm{A}}}_{4}{D}^{2}P$$

we found that

7 $${\rm{\Delta }}T={\rm{f}}({\rm{D}},{\rm{P}})={A}_{5}\,D\,P$$

where *A*_5_ is a constant. Therefore, Equation (7) reveals that the temperature difference depends linearly on droplet size and power of the heat source. This linear relation supports the previous results reported in literature^42, 43^. The essence of the equation (7) lies in that a temperature difference around a droplet can be readily estimated with a droplet size and a laser power. This is a useful strategy because directly measuring the local temperature distribution in a microchannel is difficult, especially for an opaque flow.

### Droplet trajectory shifting

**Figure 3:**
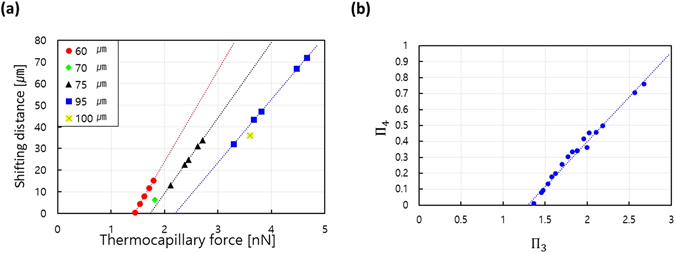
The relation between thermocapillary force and shifting distance. (**a**) The shifting distance is linearly proportional to thermocapillary force as the laser power is increased from 70 to 110 mW. The dotted lines represent a linear fit where the R^2^ value is nearly 1. (**b**) The non-dimensionalized thermocapillary force (Π_3_) and shifting distance (Π_4_) show a linear relation; all points appear on a single line with an R^2^ value of 0.9863. The slope of the dotted line is 0.5603, and the intercept of the horizontal axis occurs at 1.29.

The thermocapillary force has been applied for droplet trajectory shifting, as shown in Fig. 3a. The experimental conditions were the same as those used in the scenario depicted in Fig. 2. The shifting distance varied up to 72 μm when a force of 4.7 nN was applied to a droplet with a diameter of 95 μm. In contrast, a droplet with a diameter of 60 μm was hardly shifted with a force of 1.4 nN. To the author’s knowledge, these are the first experimental data revealing the relation between shifting distance and thermocapillary force. In addition, we found that droplet shifting could be controlled with a precision of approximately 3.4 μm over a range of 0 to 72 μm by varying the power of the heat source (laser power) by 10 mW. Note that this resolution is very fine considering that the smallest droplet has a diameter of 60 μm. This shows that the present technique, which uses repulsive thermocapillary effects, is useful for precise droplet control.

The intercept of the fitting lines on the horizontal axis in Fig. 3a indicates the minimum thermocapillary force needed to shift a droplet at a given droplet velocity. For example, the minimum force is about 2.19 nN to shift a droplet with a diameter of 95 μm and 1.4 nN for a droplet with a diameter of 60 μm. Moreover, the slopes of the fitting lines indicate the shifting distance per unit force; these are slightly but meaningfully different for different droplet sizes. The slope varies from 42.10 to 29.24 μm/nN when the droplet size is changed from 60 to 95 μm, implying that the shifting distance is also a non-linear function of droplet size. This can be attributed to the increased drag of the droplet and wall friction. It is more difficult to shift droplets with a larger drag. Also, the effects of wall friction hinder the movement of a droplet when its size approaches the size of the channel.

The above results strongly suggest that droplet shifting has the same relationships with droplet diameter and power of the heat source as does thermocapillary force. To verify this speculation, we attempted to collapse all of the data into a single line by applying Buckingham’s Pi theorem. The non-dimensional variable (*∏*_4_) for shifting distance (S) is calculated as

8 $${{\Pi }}_{4}=\frac{S\,}{D}$$

The non-dimensional relation between shifting distance and force is displayed in Fig. 3b. The data points are in good agreement with a linear fitting line, showing that the relation is linear as expected. The results of these non-dimensional analyses suggest that we can readily estimate the thermocapillary force with Equation (9) by measuring the shifting distance of a droplet, and vice versa.

9 $${{\Pi }}_{4}=0.5603({{\Pi }}_{3}-1.29)$$

This equation implies that there is a minimum force to cause the droplet shifting. Our empirical model shows that the minimum non-dimensiona force is about 1.29.

### On-demand droplet routing

**Figure 4:**
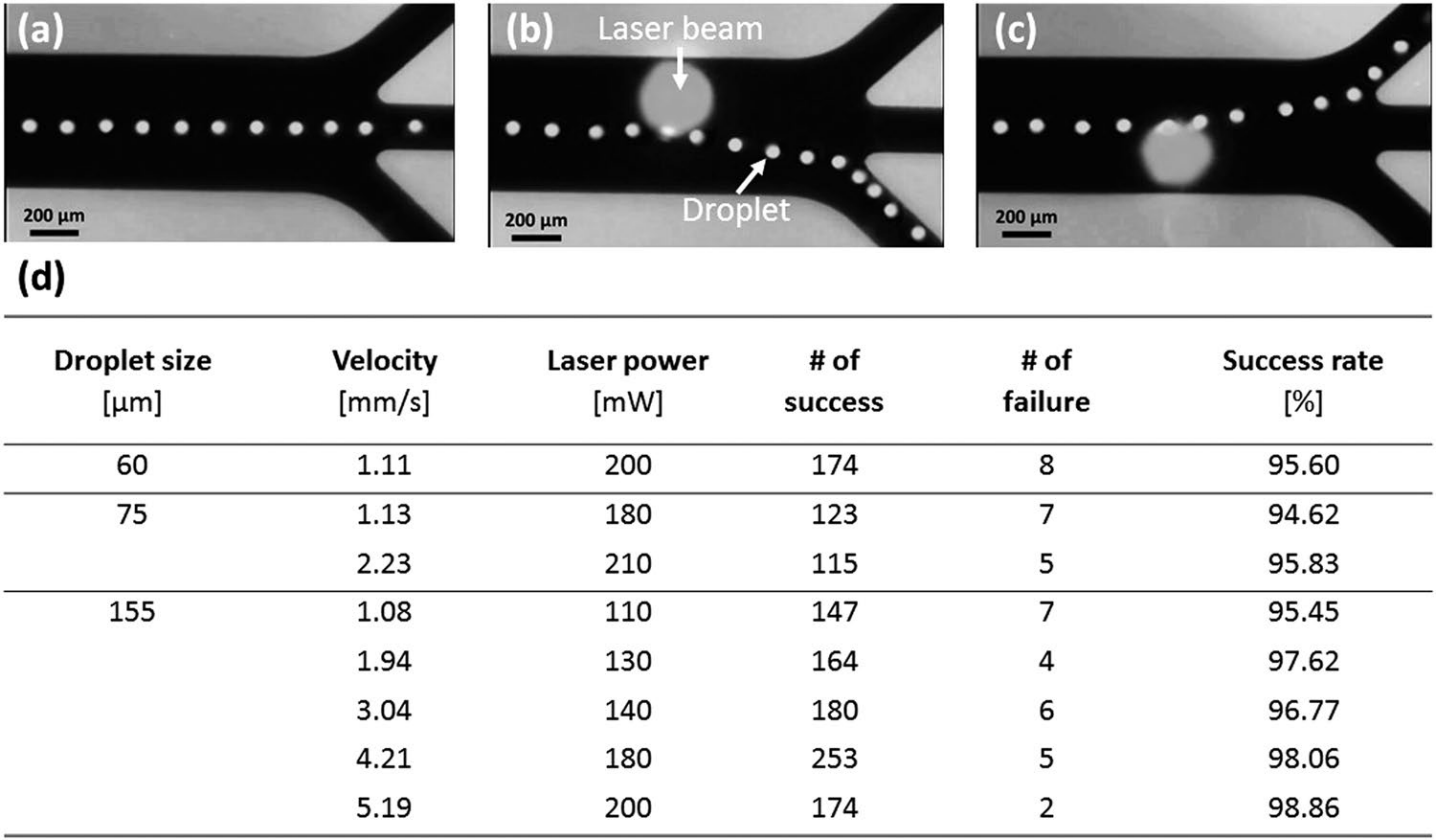
On-demand droplet routing results. Overlapping images of droplet movements (**a**) without laser beam irradiation, (**b**) with laser beam irradiation on the upper side, and (**c**) with laser beam irradiation on the lower side of the droplet trajectory. (**d**) Success rates and flow conditions of on-demand droplet routing.

An on-demand droplet routing system was developed via repulsive thermocapillary effects and on-and-off control of a laser beam on a microfluidic chip with three outlets. The on-and-off control was used to instantly maximize the temperature difference across a droplet, which consequently maximized the thermocapillary force. A droplet moving along the channel centerline was transported to the middle outlet when the laser beam was off because the three outlet channels were designed to have the same flow resistance (Fig. 4a). However, when the laser beam was irradiated off the centerline for over 0.2 sec, the droplets were transported from the opposite outlet to the laser beam spot due to repulsive thermocapillary effects (Fig. 4b and c).

We investigated the routing conditions that resulted in an at least 95% success rate by varying the droplet diameter (60, 75, and 155 μm), velocity (1.08~5.19 mm/s), and laser power level (110~210 mW), as listed in Fig. 4d. First, for a fixed laser power of 200 mW, droplet routing with a success rate of 95.60% was observed when the droplet diameter and velocity were 60 μm and 1.11 mm/s, respectively. We found that the maximum droplet velocity for successful routing increased to 5.19 mm/s with an increase in droplet diameter to 155 μm; these conditions exhibited a success rate of 98.86%. This was because the larger droplets were subject to a greater temperature difference across the droplet, resulting in stronger repulsive thermocapillary forces. Even if a higher droplet velocity leads to increased convective heat transfer, the effects of the increased droplet size appear to overwhelm the results of an increase in heat transfer, and the maximum droplet velocity for successful routing increases.

Next, for a fixed droplet size of 155 μm, the laser power required for successful routing was 110 and 200 mW for droplet velocities of 1.08 and 5.19 mm/s, respectively. This was caused by the higher velocities leading to stronger convective heat transfer and smaller temperature differences across the droplet. To maintain routing success rates above 95%, higher laser powers are necessary for higher velocities. In summary, the laser power required for successful routing decreased with a decrease in velocity and an increase in droplet size. Additionally, either a velocity below 1.11 mm/s or a laser power above 200 mW is needed to rout a droplet with a diameter smaller than 60 μm.

We expect that the present on-demand droplet routing system will be useful for cell screening assays^44, 45^. Also, we believe that the droplet control technique using thermocapillary effects will have versatile applications when the laser beam shape can be varied with a spatial light modulator or a Galvano scanner. Additionally, an algorithm for the automatic control of droplet movement should lead to a superior droplet control system. As a result, in addition to routing, droplet merging, splitting, and trapping would be possible.

## Conclusions

We studied the thermocapillary force exerted on droplets on a microfluidic chip with a dark continuous phase as the light-absorbing material. We proposed empirical relationships between force and droplet size and power of the heat source, which are measurable in a simple experimental set-up, by applying non-dimensional analysis to experimental data. Also, the droplet control of trajectory shifting and routing were implemented based on the force analysis.

In detail, the assumption of a quasi-equilibrium state between the thermocapillary and drag forces on a droplet led to a reasonable estimation of force (~nN). We found that the thermocapillary force is linearly dependent on the power of the heat source and is proportional to the square of droplet size. The shifting distance is also linearly proportional to the thermocapillary force. In addition, we could control droplet shifting up to 72 μm with a precision of 3.4 μm. Based on these results, we successfully developed an on-demand droplet routing system where the success rate was greater than 95% for various droplet sizes (60~155 μm) and velocities (1.08~5.19 mm/s).

## Materials and Methods

Indelible ink (Shachihata), used as a light-absorbing material, was mixed with oleic acid (1:4 wt. ratio), and the mixture solution was sonicated for 1 h. The interfacial tension variation between the mixture and DI water as a function of temperature was measured by a tensionmeter (Sigma 700, Attension) using the ring method. The thermal diffusivity of the ink mixtures was measured via the flash method (LFA 467 Hyper Flash, NETZSCH). The mixture viscosity was also measured by vibrating viscometer (SV-10, AND).

**Figure 5:**
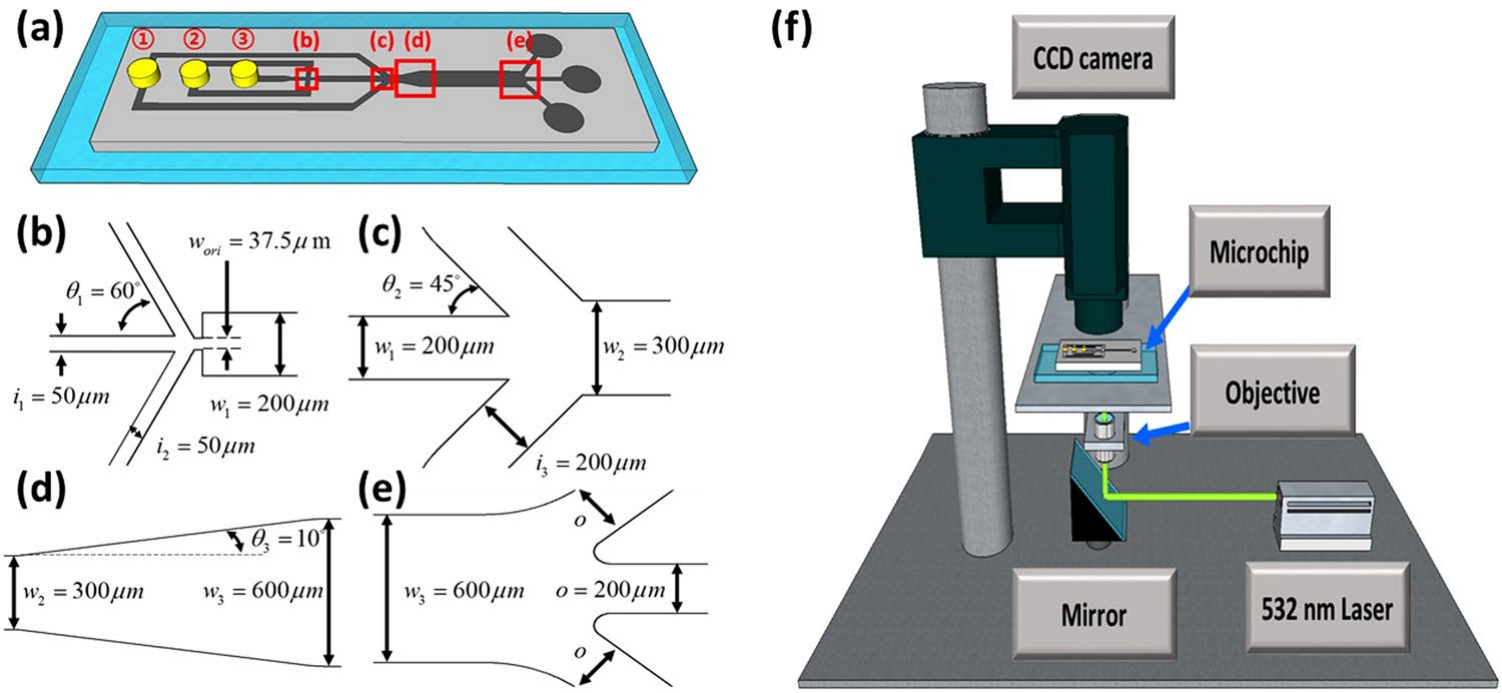
Experimental setup of (**a**) the microfluidic chip schematic with three inlets and three outlets; (**b**) the dimensions for junction 1, which is used for droplet generation; (**c**) the dimensions for junction 2, which is used to control droplet velocity and intervals; (**d**) the dimensions for expansion; (**e**) the dimensions for junction 3, which is used for droplet routing experiments; and (**f**) the experimental setup for repulsive thermocapillary effects.

A microchannel was fabricated via standard lithography and polydimethylsiloxane (PDMS) molding techniques^46–48^. This channel had three inlets and three outlets, and its height was maintained at 100 μm (Fig. 5a). The continuous phase fluid (a mixture of the ink and oleic acid) and the dispersed phase fluid (DI water) were introduced via syringe pumps (Pump 11 Plus, HARVARD APPARATUS) into inlet 2 and inlet 3, respectively. Droplets were generated at the flow focusing junction (Fig. 5b). The continuous phase fluid was additionally introduced into inlet 1 for two purposes: to control the droplet velocity and interval downstream of the second junction (Fig. 5c). The channel width was purposely expanded from 300 μm to 600 μm to secure sufficient space for a droplet to be shifted (Fig. 5d). Droplet trajectory shifting was performed in the main channel downstream of the expansion. Three outlets with a width of 200 μm were prepared to perform on-demand droplet routing (Fig. 5e). These outlet channels were designed to yield the same flow resistance.

Six different droplet diameters (60, 70, 75, 95, 100, and 155 μm) were used during the experiments. With the exception of the 155 μm droplets, all of the other droplet shapes were nearly spherical because they were smaller than the channel width and height. The 155 μm droplets were likely shaped like disks because they were larger than the channel height. The droplet speeds were controlled between the range of 1.08 and 5.19 mm/s by varying the flow rate to inlet 1. Detailed flow conditions are listed in the Supporting Information (Table S2). A 532 nm laser (LVI532CW2000FL-VA, Laserlab) was used as the heat source to generate thermocapillary effects (Fig. 5f). The laser power was varied between 70 and 220 mW, and its power was measured using a power meter (1815C, Newport). The full-width half-maximum of the laser beam was 164 μm at a power of 70 mW in the main channel. Droplet movements were monitored and recorded in real time using a CCD camera (SDC-415A, Samsung) with the aid of LabView software (National Instrument).

## Electronic supplementary material

supplementary information
