# Supplementary material for: Estimation of the thermocapillary force and its applications to precise droplet control on a microfluidic chip
Source: Sci Rep. 2017 Jun 8;7:3062. doi: 10.1038/s41598-017-03028-w (PMC5465069; doi:10.1038/s41598-017-03028-w)
Supplement: Supplementary file 1 — supplementary information [file 41598_2017_3028_MOESM1_ESM.pdf]

Estimation of the thermocapillary force and its applications to precise droplet control on a microfluidic chip

June Won[1], Wooyoung Lee[1], and Simon Song[1,2]

[1]Dept. of Mechanical Engineering, Hanyang University, Seoul, 04763, Korea
[2]Insitute of Nano Science and Technology, Hanyang University, Seoul, 04763, Korea

Supplementary Information

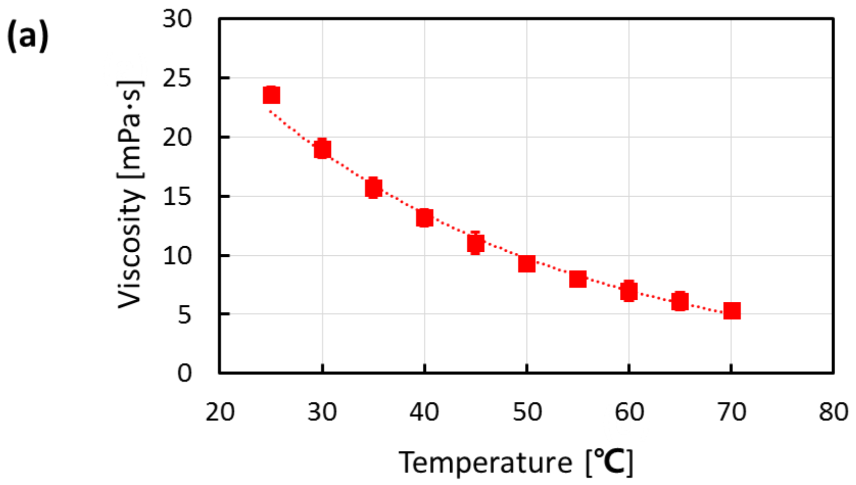

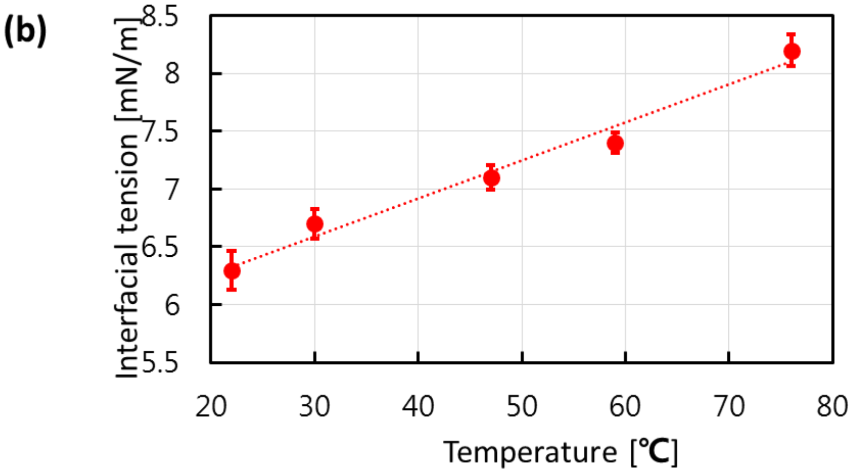

Figure S1. Ink mixture properties. (a) Relation between ink mixture viscosity and temperature. The viscosity of the mixture exponentially decreases with increasing temperature. (b) Interfacial tension variation with temperature between the ink mixture and DI water. The interfacial tension increased with an increase in temperature, implying repulsive thermocapillary effects . Each data point in both the graphs represents the average of three independent experiments. The error bars indicate a 99% confidence interval.

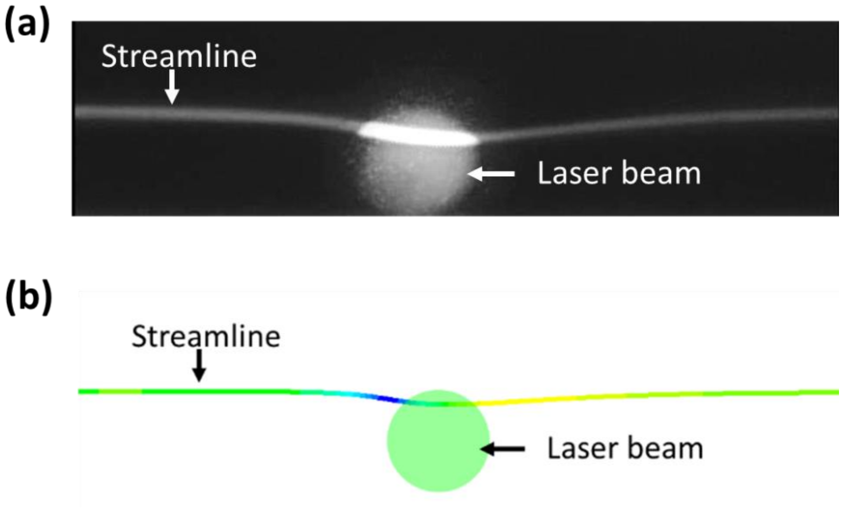

Figure S2. Streamline curvature effects determined by experiments and numerical analysis. (a) Experimentally-observed streamline curvature effects. Streamline (white line) was visualized by differentiating the concentration of the black ink (ink concentration of the black area: 20 wt % and ink concentration of the white line: 0 wt %), while the total flow rate was fixed at 17.5 μL/min. The streamline was curved toward the center of the laser beam due to local heating. (b) Numerically-observed streamline curvature effects. The second-order upwind scheme was used for this analysis, and the least squares cell-based method was used for the gradient scheme. 3,200,000 cells were used, and the meshes were polyhedral.

Calculation of repulsive thermocapillary force estimation

To estimate the repulsive thermocapillary force, a quasi-equilibrium state between repulsive thermocapillary force and drag force was assumed. Equation S1, which indicates the drag force on a spherical droplet in a rectangular channel, was used.

$$F_{drag} = 6\pi a\mu_o U \frac{1+2\mu_o/3\mu_i}{1+\mu_o/\mu_i} \qquad \text{(S1)}$$

where viscosity is a function of temperature. However, it was difficult to determine an exact value for the viscosity because the local temperature distribution was unknown. Instead, we assumed the temperature difference across the droplet based on values reported in the literature [1-3], where a typical temperature difference is about 10 ℃ for droplets of various sizes (20 ~ 100 μm). Table S1 shows the calculated values for the viscosity term of $(1+2\mu_o/3\mu_i)/(1+\mu_o/\mu_i)$ for temperature differences ranging from 0 to 70 ℃. The results show that the value is almost independent of the temperature difference. Therefore, we used 0.68 as the value for the viscosity term in the drag calculation.

Table S1. Values for the viscosity term $(1+2\mu_o/3\mu_i)/(1+\mu_o/\mu_i)$ for a range of temperature differences across a droplet.

Temperature difference [ºC]	Viscosity ($\boldsymbol{\mu_o}$) [cP]	Viscous term	Relative difference [%]
0	21.28	0.680	0.00
10	15.81	0.681	0.15
20	11.37	0.682	0.32
30	8.17	0.684	0.68
40	5.88	0.687	1.10
50	4.22	0.690	1.59
60	3.04	0.694	2.14
70	2.18	0.699	2.78

Table S2. Flow conditions used to generate droplets of different sizes. The droplet size was changed by controlling the flow rates of inlet 2 and inlet 3 (Figure 1a), and the droplet velocity and interval among droplets were varied by controlling the flow rate of inlet 1.

Droplet diameter [μm]	Droplet velocity [mm/s]	Inlet 1 flow rate [μL/min]	Inlet 2 flow rate [μL/min]	Inlet 3 flow rate [μL/min]
155	1.08	2.80	1.00	0.05
	1.94	6.50	1.00	0.05
	3.04	10.1	1.00	0.05
	4.21	13.8	1.00	0.05
	5.19	17.5	1.00	0.05
100	1.14	3.60	0.40	0.02
95	1.16	3.50	0.50	0.02
	1.73	5.25	0.50	0.02
	2.16	7.00	0.50	0.02
	2.89	8.75	0.50	0.02
	3.38	10.5	0.50	0.02
75	1.13	2.50	1.50	0.02
	2.23	6.50	1.50	0.02
70	1.11	2.40	1.60	0.02
60	1.11	2.30	1.70	0.02

References

1 Yap, Y. F. *et al.* Thermally mediated control of liquid microdroplets at a bifurcation. *J Phys D Appl Phys* **42**, doi:Artn 06550310.1088/0022-3727/42/6/065503 (2009).

2 Hu, W. & Ohta, A. T. Aqueous droplet manipulation by optically induced Marangoni circulation. *Microfluidics and Nanofluidics* **11**, 307-316 (2011).

3 Muto, M., Yamamoto, M. & Motosuke, M. A Noncontact Picolitor Droplet Handling by Photothermal Control of Interfacial Flow. *Anal Sci* **32**, 49-55, (2016).
